# Distinguishing Emotional Responses to Photographs and Artwork Using a Deep Learning-Based Approach

**DOI:** 10.3390/s19245533

**Published:** 2019-12-14

**Authors:** Heekyung Yang, Jongdae Han, Kyungha Min

**Affiliations:** 1 Industry-Academy Coorporation Foundation, Sangmyung University, Seoul 03016, Korea; yanghk@smu.ac.kr; 2 Departement of Computer Science, Sangmyung University, Seoul 03016, Korea

**Keywords:** emotion recognition, CNN, artwork, Russell model, DEAP dataset

## Abstract

Visual stimuli from photographs and artworks raise corresponding emotional responses. It is a long process to prove whether the emotions that arise from photographs and artworks are different or not. We answer this question by employing electroencephalogram (EEG)-based biosignals and a deep convolutional neural network (CNN)-based emotion recognition model. We employ Russell’s emotion model, which matches emotion keywords such as happy, calm or sad to a coordinate system whose axes are valence and arousal, respectively. We collect photographs and artwork images that match the emotion keywords and build eighteen one-minute video clips for nine emotion keywords for photographs and artwork. We hired forty subjects and executed tests about the emotional responses from the video clips. From the *t*-test on the results, we concluded that the valence shows difference, while the arousal does not.

## 1. Introduction

Emotion recognition is one of the most interesting research topics in neuroscience and human computer interface. Emotion is quantified through various emotion recognition methods that employ various bio-signals including EEG, photoplethysmography (PPG), and electrocardiography (ECG), etc. Most of the emotion recognition methods use either handcrafted features or deep learning models. The handcrafted feature-based methods recognize emotions through classical classification schemes such as support vector machine, decision tree, and principal component analysis. Recently, deep learning models such as convolutional neural network, recurrent neural network, and long and short term memory models, have been widely used since they have improved performance on emotion recognition.

Visual stimuli are known to raise corresponding emotional responses. For example, a photograph of a smiling baby give rise to a ‘happy’ emotion, and a thrilling scene such as homicide gives rise to a ‘fear’ emotion. The cause of visual stimuli comes from either photographs that capture the real world or artifactual images that are produced through various artistic media, such as pencil and brush. Emotions arisen from artwork are commonly assumed to be different from those from photographs. However, we have rarely found a quantified comparison between the emotional responses from photographs and artwork images.

In this paper, we employ EEG signals captured from human and a deep CNN structured recognition models to clearly prove that the emotional responses from photographs and artworks are different. We build an emotion recognition model from EEG signals based on a deep neural network structure and train the model using the DEAP dataset [1]. Then, we collect emotional responses from human subjects watching photographs and artwork images that convey similar contents and compare them to prove our argument that the emotion recognition from photographs and artwork images are distinguishable.

**Figure 1:**
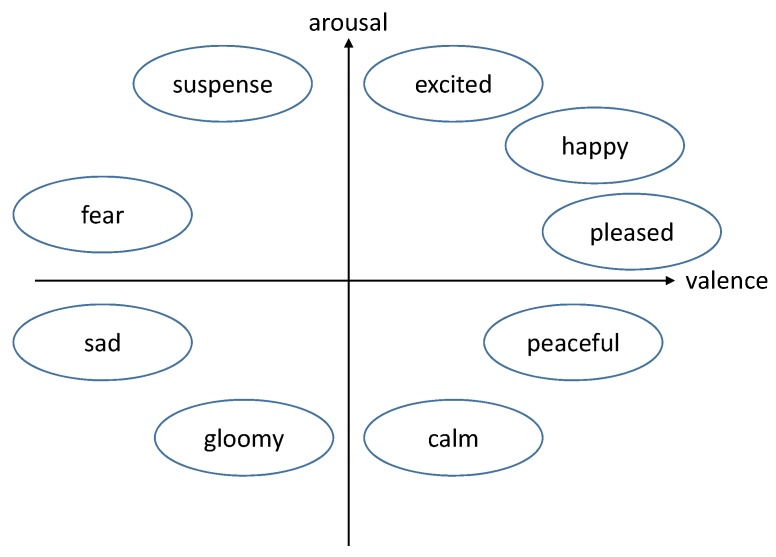
Russell’s emotion model.

We build a 2D emotion model whose *x*-axis is valence and *y*-axis is arousal. Widely-known emotion keywords such as ‘excited’, ‘happy’, ‘pleased’, ‘peaceful’, ‘calm’, ‘gloomy’, ‘sad’, ‘fear’, and ‘suspense’ are located in the 2D space according to Russell’s model, which is one of the most frequently-used emotion models (see Figure 1).

**Figure 2:**
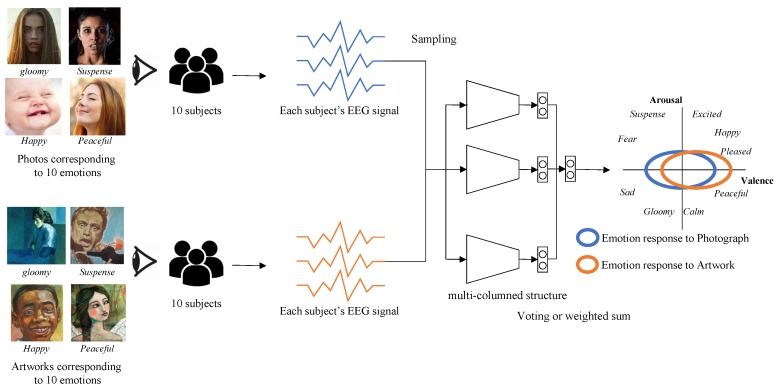
The overview of the algorithm.

Our emotion recognition model is based on a state-of-the-art multi-column convolutional neural network structure. The model is trained using the DEAP dataset, one of the most widely-used EEG big-signal datasets. The output of the model is normalized bi-modal: One for valence and the other for arousal. From the valence and arousal, we match the emotion keywords in Russell’s emotion model. The overview of our model is illustrated in Figure 2.

We hire forty human subjects and partition them into two groups: One for photographs and the other for artwork images. They watch nine one-minute long videos while their emotion is recognized from their EEG signals. The emotional response from each video is predefined. We compare the recognized emotions from both groups and compare the difference of emotional response from photographs and artwork images.

We suggest a quantitative approach to measure the differences of emotional responses from photographs and artworks. The recent progress in deep neural network research and sensor techniques presents us a set of tools to capture biosignals from users and to measure their emotional responses with high accuracy. Our work can be applied in many SNS applications. As of recent, many applications rely on various visual contents including photographs and video clips. These applications inquire whether photographs and artworks may invoke different emotional responses. If they can conclude that the artworks may invoke more positive emotional reactions than photographs, then they will focus on developing a series of filters that would render photographs into artistic styles.

This paper is organized as follows. In Section 2, we briefly review the studies on emotion recognition and the relation between visual contents and emotion. In Section 3 and Section 4, we outline the emotion recognition model and data collection process, then explain the structure of our model. We present the experiment’s details and results in Section 5 and the analysis of the results in Section 6. Finally, we draw conclusions of our work and suggest a future plan in Section 7.

## 2. Related Work

In this section, we describe previous studies around deep learning, emotion recognition approaches and emotional responses from visual contents.

### 2.1. Deep Learning-Based Emotion Recognition Studies

#### 2.1.1. Early Models

Early studies including [2,3,4,5,6,7,8] utilized handpicked features and applied various machine learning techniques such as support vector machine and principal component analysis (PCA) to the features. While these studies show viable accuracy, performances are restricted from feature selection.

To resolve the restriction, many studies adopted deep learning without feature selection. Jirayucharoensak et al. [9] developed a model for recognizing emotions from nonstationary EEG signals. Stacked autoencoder (SAE) is adopted to learn the features extracted from EEG signals. Additionally, they applied principal component analysis (PCA) that extracts the most important components and covariate shift adaptation in order to minimize the nonstationary effect; this also leads to less overfitting.

Khosrowabadi et al. [10] developed a multilayer feedforward neural network for discriminating human emotions from unstationary EEG signals. The network is a composition of spectral filtering layers that analyze input signals and a shift register memory layer. They discriminate the emotion from EEG signals and represents it with valence and arousal levels, according to Russell’s model.

#### 2.1.2. CNN-Based Models

Earlier approaches had the common restriction that features had to be picked by a human expert. With the evolution of convolution neural networks, many studies utilize CNN to eliminate this restriction.

Tang et al. [11] built a deep CNN-based classification model for motor imagery. The model had F-scores of 87.76∼86.64% for classification of motor imagery for left and right hands. While the performance of the model is not significantly better than conventional hand-picked feature-based models, the model has shown viability of multi-layer CNN for EEG recognition.

Croce et al. [12] deployed a CNN model for classification of brain and artifactual independent components. While the study is not directly related to emotion recognition, independent component analysis is essential for the classic feature selection approach. They show that high CNN classification performances (92.4% for EEG) were achieved through heuristical selection of machinery hyperparameters and through the CNN self-selection of the features of interest

Tripathi et al. [13] developed a CNN-based model that recognizes emotions from EEG signals. The DEAP dataset is used to train two different neural network models: A deep neural network with simpler layers and a convolutional neural network. According to their experiment, the CNN-based model demonstrated an improved performance of 4.96% more than the existing, simple layer-based models. The study has shown the possibility of CNN upon EEG signal analysis.

Salama et al. [14] proposed a 3D CNN model to recognize emotions from EEG signals of multi-channel structure. EEG signals have a spatio-temporal aspect, hence 3D CNN is appropriate to train with the signals. Moreover, a data augmentation phase is introduced to improve the accuracy of the model, possibly reducing overfitting. They tested that their model with a DEAP dataset and recorded accuracies of 88.49% for arousal and 87.44% for valence.

Moon et al. [15] presented a CNN-based emotion recognition model for EEG signals. A major difference of their approach is its brain connectivity features, used to account for synchronous activation of many different brain regions. For this reason, their method can effectively capture various asymmetric brain activity patterns that play a key role in emotion recognition. While their approach does not solely rely on CNN, they show the CNN-based approach can be improved with partial feature selection.

Yang et al. [16] presented a multi-channel structured-CNN model to recognise emotions from unstationary EEG signals. Their model is composed of several independent recognition modules, which are designed based on the DenseNet model [17]. The individual decisions of the modules are merged to build a final decision of the model. They employed the DEAP dataset for a comparison, and showed an excellent accuracy for valence and arousal.

Zhang et al. [18] presented an effective spatial attention map (SAM) to weigh the multi-hierarchical convolutional features. SAM help reduce the filter values corresponding to background features. A multi-model adaptive response fusion (MAF) mechanism is also presented for adaptive weighted fusion of multiple response maps generated by attentional feature.

#### 2.1.3. RNN-Based Models

Recurrent neural network (RNN), which is known to be effective in processing time series data, has been employed for recognizing emotions from EEG signals which are definitely time series.

Alhagry et al. [19] presented a long-short term memory (LSTM) RNN-based model that recognizes emotions from unstationary EEG signals. Their model recognizes emotions in three axes: Arousal, valence and liking. As a result, they recorded accuracy of greater than 85% for the three axes of emotion.

Li et al. [20] developed an RNN-based emotion recognition model. The model considers three characteristics of EEG signals: Temporal, spatial and frequency. Their model extracts rational asymmetry (RASM) to describe the frequency and spatial domain characteristic of EEG signals. The last characteristic, temporal correlation, is explored by an LSTM RNN model. They tested their model using the DEAP dataset and demonstrated 76.67% mean accuracy.

Xing et al. [21] presented an emotion recognition framework using multi-channel EEG. A linear EEG mixing model and an emotion time model composes their framework, which decomposes the EEG source signals from the collected EEG signals and improves classification accuracy. The EEG mixing model is based on a stacked auto encoder (SAE) and the emotion timing model is based on LSTM RNN. As a result, their framework achieved accuracies of 74.38% for arousal and 81.10% for valence with the DEAP dataset.

#### 2.1.4. Hybrid Models

As in other domains, deep learning-based models can be improved with traditional feature-selection models if appropriate. Several studies integrate deep learning-based models into the other machine learning techniques, while others utilize multiple different deep learning-based models.

Yang et al. [22] presented a hybrid neural network-based model that combines CNN and RNN to classify human emotions. They consider baseline signals as an effective pre-processing method to improve the recognition accuracy, like in traditional feature-based approaches. On the other hand, their model learns spatio-temporal representation of unstationary EEG signals for emotion classification. The CNN module estimates inter-channel correlations among physically adjacent EEG signals, while The RNN module extracts contextual information of the signals. They tested the model with the DEAP dataset and demonstrated accuracies of 91.03% for arousal and 90.80% for valence.

Yoo et al. [23] developed a neural network-based model that recognizes six emotional states including joy, happiness, fear, anger, despair, and sadness. They designed their model using an artificial neural network and tested various multi-model bio-signals including EEG, ECG, PPG, and GSR for emotion recognition.

### 2.2. Emotional Response from Visual Contents

Kim and Kim [24] investigated the sensitivity preference perceived differently according to the difference in color temperature. They extracted six pairs of words such as warm–cold, tender–wild, dynamic–static, complicated–simple, light–heavy, and positive–negative from various expressions on colors and surveyed fifty human subjects whether the emotions in the color temperature range matched these words. This investigation reveals that some emotional expressions from human subjects are located in a specific range of the color temperature.

**Figure 3:**
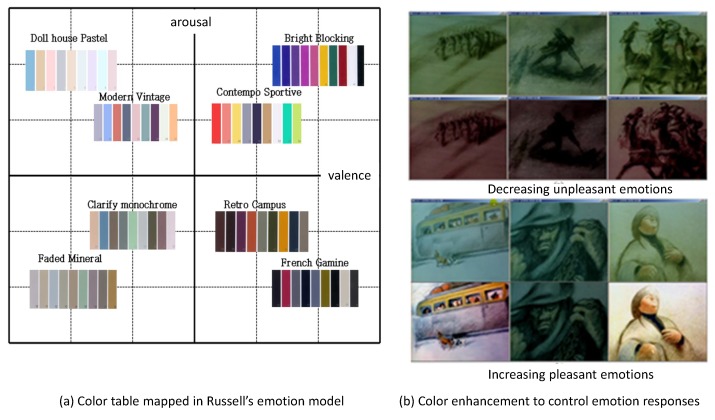
Examples from related works.

Lechner et al. [25] studied the relationships between color and emotion in various industries. They recognized the mismatch between the existing color table for color designers and the emotions sensed by users. They surveyed ninety focus groups in related fields to build the relationship between color tables and emotions. As a result, they presented a group of colors that match the emotions located in Russell’s emotion model. They also named each group of colors according to the frequently used terminologies in the fashion industry, their color model is very practical to many experts in the fashion and design industry (see Figure 3a).

Yang et al. [26] presented an emotion enhancement scheme by controlling the color of visual contents. They arranged the color table in Russell’s model and modified the colors of a famous animation to enhance the emotional responses from users. They executed a user study that proves the effectiveness of their approach. One group watches a video clip from the original animation, and the other group watches a different video clip re-rendered by applying their color enhancement scheme to the original animation. The emotional responses from the latter group are clearly distinguished from the former group (see Figure 3b).

## 3. Emotion Model

### 3.1. Russell’s Model

Russell [27] presented a classical emotion model that decomposes an emotion into two axes: Valence and arousal. In this model, valence and arousal are independent terms. Therefore, valence does not influence arousal, and vice versa. In this model, an emotion such as sadness, suspense, excitement, or happiness is decomposed into a pair of two values (valence and arousal). This pair is located in a 2D space whose **x**-axis corresponds to valence and *y*-axis to arousal (see Figure 1).

### 3.2. Emotion Dataset Construction

**Figure 4:**
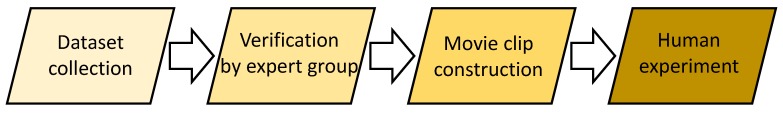
Our emotion dataset construction process.

Our emotion dataset is constructed in the process illustrated in Figure 4.

#### 3.2.1. Dataset Collection

**Figure 5:**
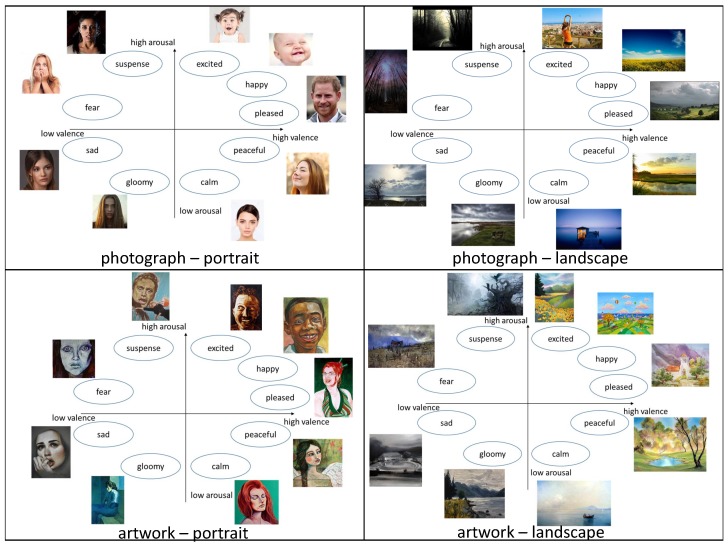
Sampled images of our dataset.

In our approach, we select nine emotions and map them into Russell’s model. The emotion keywords we selected are ‘excited’, ‘happy’, ‘pleased’, ‘peaceful’, ‘calm’, ‘gloomy’, ‘sad’, ‘fear’, and ‘suspense’. To build a dataset for these emotion keywords, we searched webpages with these keywords and collected images. We built two datasets: One for photographs and the other for artwork images. We also categorized the images into two groups: Portraits and landscapes. A sample of our dataset is illustrated in Figure 5. We collect five portraits each for both photographs and artwork, and five landscapes each for both photographs and artwork.

#### 3.2.2. Verification by Expert Group

To verify that the images in our dataset are correctly bound to the emotion keyword we have aimed at, we collected an expert group composed of eight experts: Three game designers, two cartoonists and three animation directors. They were asked to pair the images to the most proper emotion keyword among the nine emotion keywords. After they paired the images to emotion keywords, we accepted images which were paired to an identical keyword by more than six experts. For the discarded images, we reselected candidate images and tested them again until they were paired to a target keyword by more than six experts. The dataset is presented in Appendix A.

#### 3.2.3. Movie Clip Construction

We built eighteen one minute-length video clips from the images in our dataset. In each movie clip, ten images of the same emotion keyword are played for six seconds. Nine clips were produced from photographs and another nine clips from artwork. Each movie clip was paired to an emotion keyword. For example, a movie clip was named ‘photo–happy’ or artwork–sad.

## 4. Emotion Recognition Model

### 4.1. Multi-Column Model

**Figure 6:**
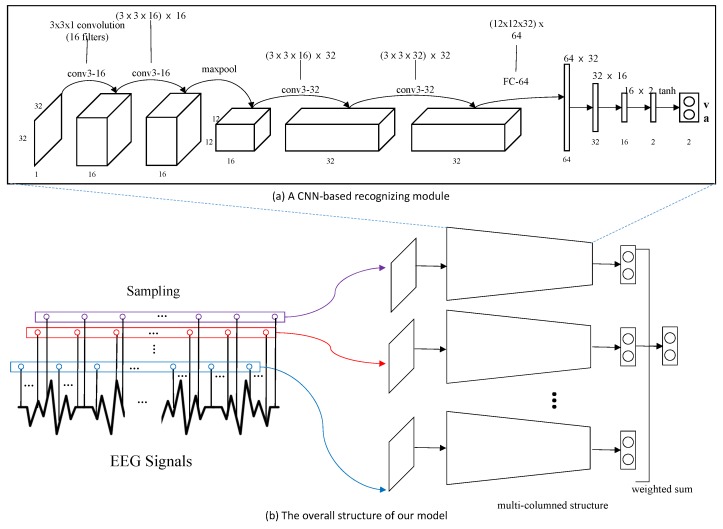
The structure of our emotion recognition model.

We employ a multi-column structured model, which shows state-of-the-art accuracy in recognizing emotion from EEG signals [16]. This model is composed of several recognizing modules that process the EEG signal independently. Each recognizing module is designed using a CNN structure, which is illustrated in Figure 6a. We sample 1024 samples from EEG signals for one minute and reorganize them into 32 × 32 rectangular form. The EEG signal in rectangular form is fed into each recognizing module of our model and the decisions from the individual modules are merged to form the final decision of our model. We apply a weighted average scheme to the individual decisions ($v_{i}$) for the final decision ($v_{final}$) as follows:

$$v_{final}=\frac{\sum_{i=1}^{k}w_{i}v_{i}}{\sum_{i=1}^{k}w_{i}},$$

where $v_{i}$ is a binary value having +1 or –1 and $w_{i}$ is the predicted probability of the *i*-th module.

The whole structure of our model is illustrated in Figure 6b. According to [16], the best accuracy is achieved for a model of five recognizing modules. Therefore, we built our model with five recognizing modules.

### 4.2. Model Training

To train our model, we employed the DEAP dataset, which is one of the most frequently used EEG signal dataset. For the 32 participants of the DEAP dataset, we selected EEG signals from 22 participants for training dataset, 5 for validation and 5 for test. Each participant executed 40 experiments. We decomposed the EEG signal from a participant by sampling 32 values from 7680/(32×k) different positions, where we set *k* to 5.

*k* is the number of recognition modules in our multi-column emotion recognition model. Yang et al. [16] tested four values for *k*: 1, 3, 5 and 7. Among the four values of *k*, k=5 shows the highest accuracy for valence and the second highest accuracy for arousal. Furthermore, k=5 shows faster computation time than k=7, which shows the second highest accuracy for valence and the highest accuracy for arousal. Therefore, we select *k* as 5.

From this strategy, we collect 33∼80 EEG training data for a participant of a video clip, and this leads to a training dataset of 29,040∼70,400. Similarly, we built datasets of 6600 ∼16,000 for the validation and test sets, respectively.

For training, we set the learning rate of our model as 0.0001, which is decreased by 10 times according to the decrease of an error in a validation dataset. The weight decay is assigned as 0.5 and the batch size as 100. The training process takes approximately 1.5 hours.

For training, we recorded 95.27% accuracy for valence and 96.19% for arousal. For test, we recorded 90.01% and 90.65% accuracies for valence and arousal, respectively. Based on these accuracies, we decided to employ the multi-column model in [16], which is one of the state-of-the-art EEG-based emotion recognition models, for our study. We did not need to retrain this model in this study, since the training of the model was already finished.

## 5. Experiment and Result

In this section, we describe details about our experiment and its result. Our research goal is to find out the difference of intensity and/or class for emotional response against artwork and photographs.

### 5.1. Experiment Setup

As described before, our emotion recognition model is trained with a DEAP dataset. Therefore, it is assumed our experiment setup was prepared as similar to that of DEAP. While the DEAP experiment utilizes 16 auxiliary channels beside 32 EEG channel, our experiment excluded those channels because our emotion recognition model solely depends on EEG channels.

We use LiveAmp 32 and LiveCap [28], which allow us to set up 32 channels following a standard 10/20 system [29]. Our participants are asked to watch eighteen one-minute videos, and recordings are converted to .EEG files. Time of start and end of each playback is required to keep track of the experiment and synchronize EEG and playback events. Because of differences between our equipment and that of the DEAP experiment, We took extra precautions with our preprocessing. We deployed event markers during our recordings to slice them precisely. Table 1 shows these markers and their descriptions.

Each participant was asked to watch our videos under our surveillance. We controlled both the start of the recording and playback, and recorded each starting time. We used 3 s of baseline recording, then started playback.

### 5.2. Experiment

Recordings from forty participants are converted into the widely-used .EEG format using the proprietary converter from LiveAmp. We use EEGLab with MATLAB to preprocess those recordings alongside the channel location file. Detailed info about the preprocessing is as follows:

- The data was downsampled to 128 Hz.
- A bandpass frequency filter from 4.0–45.0 Hz was applied.
- The EEG channels were reordered so that they all follow that of DEAP.
- The data was segmented into eighteen 60-second trials and one baseline recording.
- Detrending was performed.

In general, the goal of the preprocessing is to streamline our results with the DEAP dataset. Preprocessed results were put into our multi-column emotion recognition model, and the model estimated valence and arousal values for each result.

### 5.3. Result

We hired forty subjects for our experiment: Twenty for photographs and twenty for artwork. We hired forty subjects to average out the difference of the emotional reactions on photographs and artworks that may come from the personal preferences of the subjects. Furthermore, we also carefully checked the age, sex and background of the subjects to decrease the personal differences. The distributions of the subjects for age, sex and background are shown in Table 2.

**Figure 7:**
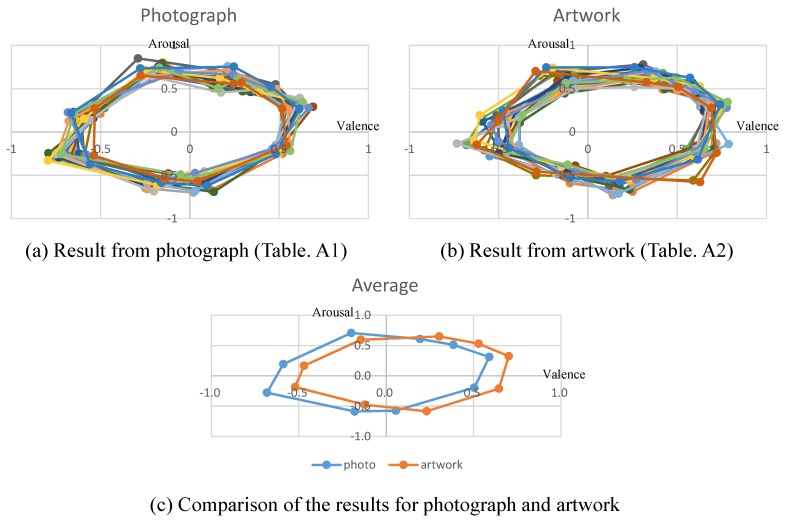
The result of our experiment.

Their responses for valence and arousal are presented in Table A1 and Table A2. These values are illustrated in Figure 7a,b. In Figure 7c, we compare the average responses from photographs and from artwork, respectively. The graphs in Figure 7c show the difference of the emotional responses from photographs and artwork.

## 6. Analysis

### 6.1. Quantitative Analysis Through t-Test

To analyze the results from our experiment, we built a hypothesis that the valence from the group watching artwork images increases compared to the valence from the group watching photographs. Therefore, the purpose of this analysis is to prove that the valences from the nine emotional keywords are different for the artwork group and photograph group. We apply the *t*-test for the nine emotion keywords and estimate *p* value. The *p* values of two groups for emotion keywords are shown in Table 3.

According to the *t*-test, we conclude that the difference of the valence for ‘gloomy’ and ‘suspense’ is significant at a confidence level of p<0.05 and the difference of the valence for other emotion keywords is significant at a confidence level of p<0.01. Furthermore, the difference of the arousal for ‘gloomy’, ‘sad’, and ‘suspense’ is significant at a confidence level of p<0.01 and the difference of the arousal for other emotion keywords is not significant.

### 6.2. Further Analysis

In our quantitative analysis, the emotional responses from artworks show greater valence than the responses from photographs, while the arousal is not distinguishable. From this analysis, we conclude that the magnitude for the responses of pleasant emotions such as ‘excited’, ‘happy’, ‘pleased’, ‘peaceful’, and ‘calm’ increases, while the magnitude for unpleasant emotions such as ‘gloomy’, ‘sad’, ‘fear’, and ‘suspense’ decreases.

**Figure 8:**
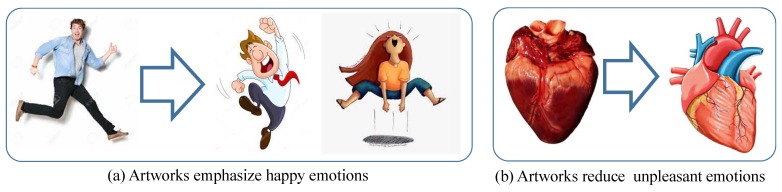
The increase of valence in artwork results in different effects: Increase of happy emotion and decrease of unpleasant emotion.

We find that many artworks that represent positive emotions such as excited or happy are exaggerated. In Figure 8a, the happy actions in artworks to the left of the arrow represent some actions that would not exist in the real world. Such an exaggeration can be a reason of our result that the valence of positive emotions from artworks becomes higher than the valence of positive emotions from photographs. We also admit that users may feel happier emotion from photographs of a baby’s smile than artworks of a baby’s smile. However, the efforts of artists to exaggerate positive emotions can increase the valence responses from artworks.

On the other hand, artists tend to reduce unpleasant emotions from scenes they draw. Furthermore, the artistic mediums such as pigment from pencil or brush convey less unpleasant emotions than real scenes. We can observe the reduction of unpleasant scenes in some practical illustrations such as anatomical illustrations (see Figure 8b). To decrease the unpleasant feelings from real objects, artistic illustration is employed.

The type of artwork such as portrait or landscape does not affect the emotional responses from human subjects.

### 6.3. Limitation

In our analysis, valence shows a meaningful difference, but the arousal does not. Our study has a limitation in that it cannot specify the reason why the arousal does not show a meaningful difference. The scope of our study is to measure the difference of emotional reactions for photographs and artwork. Measuring the time required for an emotional reaction may be required to analyze the difference of arousal for photographs and artworks.

## 7. Conclusions and Future Work

In this paper, we attack the question whether the visual stimuli that come from photographs and artwork are different or not, using an EEG-based biosignal and multi-column structured emotion recognition model. We employ Russell’s emotion model, which matches emotion keywords to valence and arousal. Various photographs and artwork images that match nine emotion keywords were collected to build eighteen video clips for test with humans. Forty subjects in two groups watched the video clips and the emotions from the subjects were recognized through EEG signals and our emotion recognition model. The *t*-test on the results shows that valence in the two groups is different, while the arousal is not distinguishable.

As visual content such as photos and video clips is widely used in social networks, many social network service companies try to improve the visual satisfaction of their visual contents by evoking emotional reactions from users. Therefore they present many filters that enhance the emotions embedded in their visual contents. We assume that the result of our paper can present theoretical backgrounds for this trend. Even though it is hard to convert a photograph into artwork, many filters that endow photographs with the feeling of artwork can enhance or enfeeble the emotional reactions from the photographs.

## Figures and Tables

**Table 1:** Index for event markers.

Marker Code	Event Description	Estimated Event Length
1	start of baseline recording	(3000 ms)
2	start of video playback 1	60,000 ms
3	end of video playback 1	5000 ms
4	start of video playback 2	60,000 ms
...	...	...
35	end of video playback 17	5000 ms
36	start of video playback 18	60,000 ms
37	end of video playback 18	5000 ms
38	end of recording	-

**Table 2:** The distributions of the subjects.

		Photograph Group	Artwork Group
	in 20 s	10	12
age	in 30 s	6	5
	in 40 s or older	4	3
	sum	20	20
	female	9	10
sex	male	11	10
	sum	20	20
	engineering and science	8	6
	social science	7	8
background	art	3	4
	other	2	2
	sum	20	20

**Table 3:** *p* Values for the valences and arousals of nine emotion keywords.

	Excited	Happy	Pleased	Peaceful	Calm	Gloomy	Sad	Fear	Suspense
valence	4.59 × 10^−8^	7.89 × 10^−9^	4.66 × 10^−9^	7.79 × 10^−10^	1.45 × 10^−5^	3.70 × 10^−3^	2.24 × 10^−6^	1.58 × 10^−6^	6.33 × 10^−4^
arousal	1.02 × 10^−1^	1.34 × 10^−1^	2.48 × 10^−1^	2.51 × 10^−1^	6.13 × 10^−1^	6.88 × 10^−8^	4.25 × 10^−8^	7.32 × 10^−2^	3.38× 10^−5^

**Figure A1:**
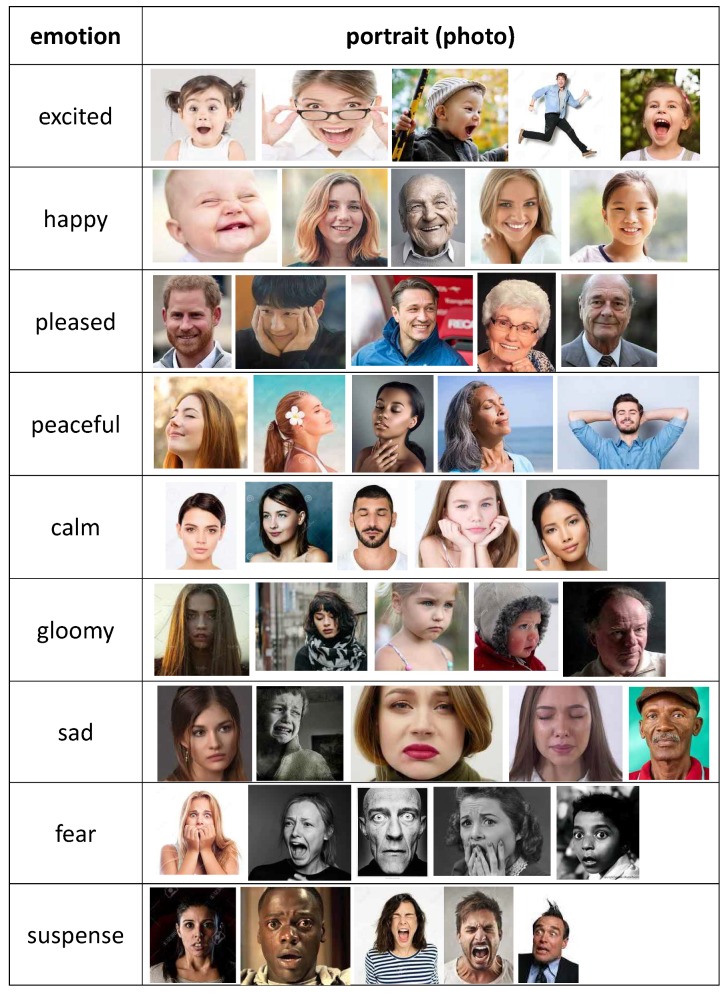
Dataset for photographic portrait.

**Figure A2:**
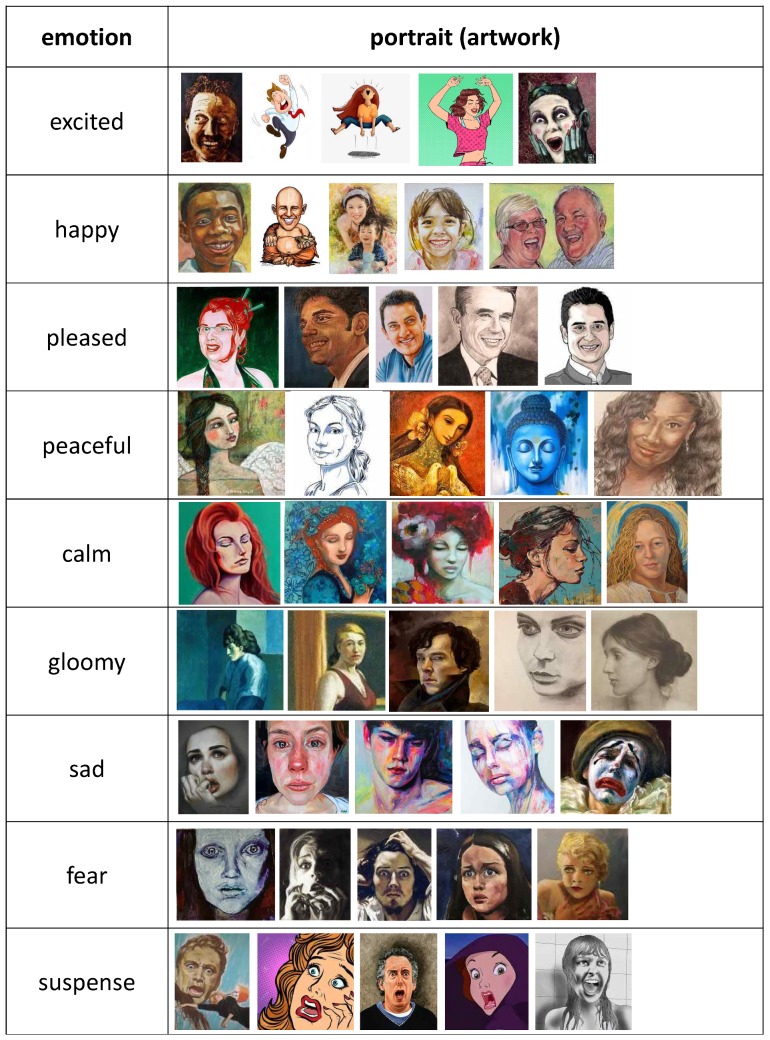
Dataset for artwork portrait.

**Figure A3:**
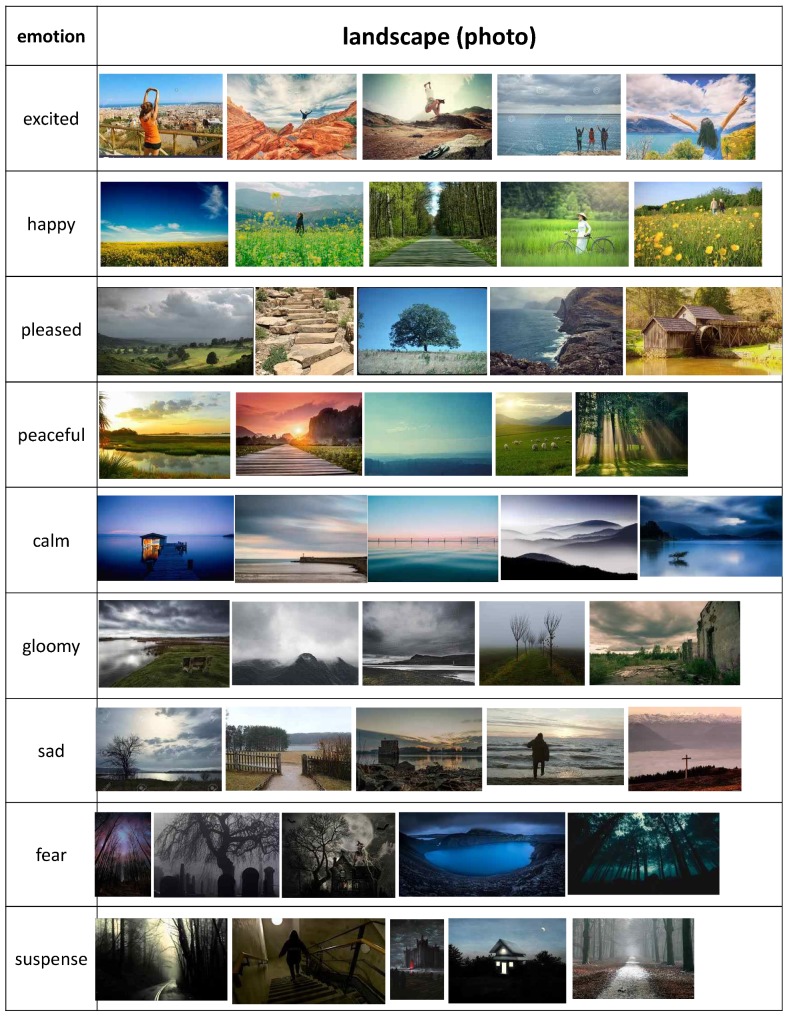
Dataset for photographic landscape.

**Figure A4:**
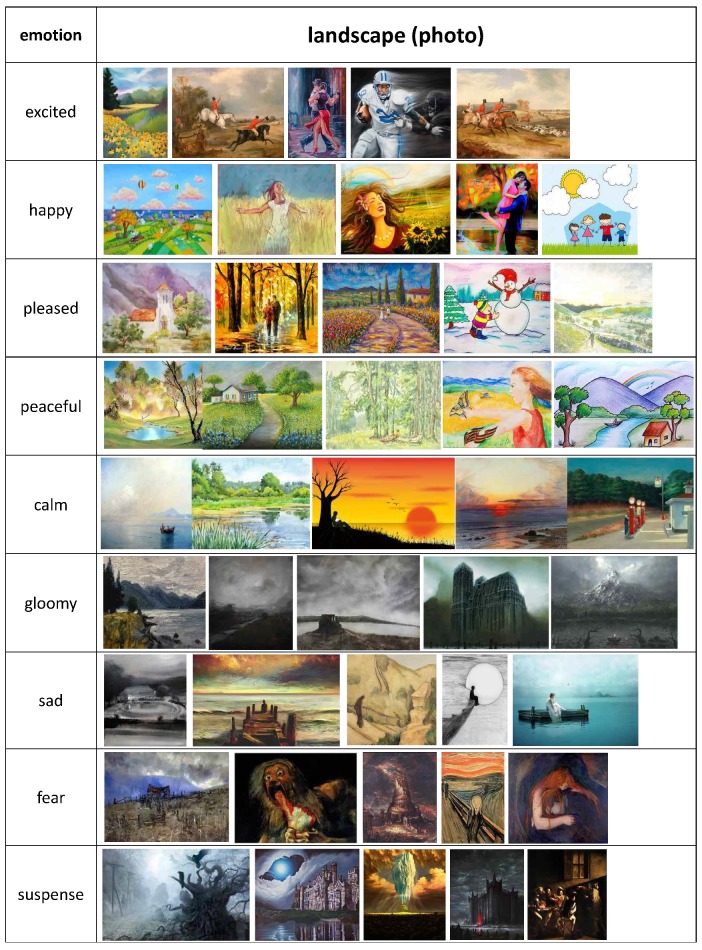
Dataset for artwork landscape.

## Appendix A

This appendix presents the table for the user test and the images in the dataset we employed for our study. It contains photographic portrait in Figure A1, artwork portrait in Figure A2, photographic landscape in Figure A3 and artwork landscape in Figure A4.

**Table A1:** The emotions estimated from twenty subjects for photograph (Val. for valence and Arou. for arousal).

		Excited	Happy	Pleased	Peaceful	Calm	Gloomy	Sad	Fear	Suspense
sub1	Val.	0.2	0.4	0.6	0.5	0.1	−0.2	−0.7	−0.6	−0.2
	Arou.	0.6	0.5	0.3	−0.2	−0.6	−0.6	−0.3	0.2	0.7
sub2	Val.	0.15	0.33	0.58	0.52	0.12	−0.17	−0.75	−0.5	−0.15
	Arou.	0.5	0.47	0.32	−0.52	−0.67	−0.62	−0.25	0.21	0.8
sub3	Val.	0.21	0.46	0.53	0.52	0.02	−0.25	−0.65	−0.58	−0.17
	Arou.	0.71	0.51	0.28	−0.15	−0.7	−0.65	−0.27	0.15	0.65
sub4	Val.	0.18	0.42	0.59	0.51	0.07	−0.21	−0.68	−0.62	−0.21
	Arou.	0.65	0.51	0.31	−0.17	−0.58	−0.61	−0.29	0.17	0.61
sub5	Val.	0.19	0.41	0.57	0.53	0.09	−0.22	−0.71	−0.57	−0.19
	Arou.	0.52	0.49	0.33	−0.17	−0.51	−0.62	−0.22	0.16	0.65
sub6	Val.	0.22	0.45	0.59	0.48	0.05	−0.23	−0.75	−0.59	−0.18
	Arou.	0.59	0.51	0.35	−0.21	−0.55	−0.59	−0.31	0.19	0.75
sub7	Val.	0.23	0.42	0.61	0.52	0.05	−0.16	−0.62	−0.65	−0.13
	Arou.	0.63	0.52	0.32	−0.19	−0.53	−0.57	−0.25	0.23	0.67
sub8	Val.	0.17	0.3	0.69	0.52	0.03	−0.12	−0.67	−0.59	−0.21
	Arou.	0.54	0.58	0.29	−0.15	−0.49	−0.48	−0.31	0.19	0.7
sub9	Val.	0.21	0.48	0.59	0.45	0.05	−0.12	−0.59	−0.61	−0.29
	Arou.	0.7	0.55	0.32	−0.25	−0.61	−0.55	−0.32	0.21	0.85
sub10	Val.	0.2	0.31	0.51	0.52	0.05	−0.14	−0.59	−0.54	−0.21
	Arou.	0.59	0.52	0.31	−0.19	−0.55	−0.54	−0.27	0.23	0.71
sub11	Val.	0.13	0.34	0.63	0.47	0.06	−0.24	−0.73	−0.54	−0.17
	Arou.	0.59	0.46	0.33	−0.18	−0.59	−0.63	−0.30	0.16	0.76
sub12	Val.	0.13	0.29	0.55	0.49	0.13	−0.21	−0.79	−0.48	−0.16
	Arou.	0.51	0.47	0.34	−0.21	−0.69	−0.60	−0.25	0.28	0.79
sub13	Val.	0.21	0.39	0.54	0.48	0.03	−0.24	−0.72	−0.55	−0.21
	Arou.	0.76	0.48	0.28	−0.15	−0.68	−0.59	−0.24	0.14	0.69
sub14	Val.	0.21	0.49	0.55	0.56	0.02	−0.24	−0.74	−0.68	−0.26
	Arou.	0.72	0.50	0.26	−0.14	−0.54	−0.66	−0.24	0.12	0.65
sub15	Val.	0.17	0.37	0.62	0.46	0.08	−0.20	−0.75	−0.56	−0.21
	Arou.	0.46	0.49	0.39	−0.22	−0.46	−0.69	−0.26	0.14	0.64
sub16	Val.	0.17	0.39	0.64	0.50	−0.01	−0.18	−0.79	−0.59	−0.25
	Arou.	0.62	0.46	0.33	−0.25	−0.50	−0.61	−0.33	0.15	0.72
sub17	Val.	0.24	0.43	0.67	0.46	0.03	−0.12	−0.58	−0.68	−0.06
	Arou.	0.66	0.51	0.28	−0.20	−0.48	−0.54	−0.26	0.23	0.69
sub18	Val.	0.16	0.27	0.63	0.56	−0.01	−0.05	−0.72	−0.65	−0.17
	Arou.	0.51	0.60	0.34	−0.22	−0.55	−0.49	−0.26	0.22	0.74
sub19	Val.	0.25	0.45	0.61	0.48	0.10	−0.16	−0.56	−0.66	−0.28
	Arou.	0.75	0.52	0.27	−0.26	−0.62	−0.59	−0.37	0.22	0.73
sub20	Val.	0.25	0.29	0.52	0.54	0.04	−0.14	−0.54	−0.53	−0.28
	Arou.	0.56	0.57	0.27	−0.16	−0.57	−0.54	−0.27	0.27	0.65
average	Val.	0.194	0.385	0.590	0.504	0.055	−0.180	−0.682	−0.589	−0.200
	Arou.	0.608	0.510	0.312	−0.195	−0.574	−0.588	−0.278	0.193	0.708

**Table A2:** The emotions estimated from twenty subjects for artwork (Val. for valence and Arou. for arousal).

		Excited	Happy	Pleased	Peaceful	Calm	Gloomy	Sad	Fear	Suspense
sub21	Val.	0.32	0.53	0.67	0.61	0.18	−0.13	−0.55	−0.47	−0.14
	Arou.	0.67	0.52	0.31	−0.21	−0.61	−0.15	−0.28	0.21	0.59
sub22	Val.	0.26	0.42	0.71	0.69	0.25	−0.1	−0.45	−0.45	−0.09
	Arou.	0.61	0.49	0.33	−0.25	−0.69	−0.59	−0.21	0.15	0.49
sub23	Val.	0.33	0.53	0.63	0.72	0.14	−0.08	−0.45	−0.39	−0.07
	Arou.	0.72	0.49	0.29	−0.13	−0.73	−0.49	−0.19	0.2	0.6
sub24	Val.	0.21	0.52	0.69	0.62	0.19	−0.08	−0.47	−0.42	−0.1
	Arou.	0.61	0.52	0.35	−0.18	−0.52	−0.48	−0.21	0.15	0.52
sub25	Val.	0.29	0.51	0.69	0.62	0.21	−0.17	−0.68	−0.52	−0.1
	Arou.	0.59	0.54	0.34	−0.19	−0.55	−0.49	−0.15	0.14	0.55
sub26	Val.	0.42	0.61	0.71	0.55	0.19	−0.1	−0.68	−0.17	0.42
	Arou.	0.68	0.53	0.35	−0.2	−0.53	−0.45	−0.14	0.68	0.67
sub27	Val.	0.33	0.56	0.72	0.65	0.23	−0.11	−0.51	−0.1	0.33
	Arou.	0.71	0.54	0.35	−0.21	−0.52	−0.49	−0.15	0.59	0.71
sub28	Val.	0.29	0.49	0.75	0.61	0.1	−0.07	−0.5	−0.17	0.29
	Arou.	0.65	0.59	0.3	−0.16	−0.51	−0.39	−0.25	0.59	0.65
sub29	Val.	0.31	0.54	0.69	0.55	0.12	−0.09	−0.49	−0.19	0.31
	Arou.	0.78	0.56	0.34	−0.27	−0.62	−0.48	−0.21	0.69	0.78
sub30	Val.	0.39	0.48	0.65	0.72	0.59	−0.29	−0.58	−0.27	0.39
	Arou.	0.62	0.53	0.32	−0.21	−0.56	−0.5	−0.21	0.65	0.62
sub31	Val.	0.29	0.59	0.65	0.65	0.17	−0.11	−0.49	−0.41	−0.11
	Arou.	0.74	0.56	0.24	−0.20	−0.59	−0.43	−0.22	0.23	0.65
sub32	Val.	0.26	0.43	0.71	0.71	0.23	−0.07	−0.38	−0.38	−0.13
	Arou.	0.57	0.50	0.35	−0.19	−0.66	−0.53	−0.23	0.11	0.45
sub33	Val.	0.38	0.51	0.63	0.79	0.17	−0.08	−0.39	−0.38	−0.08
	Arou.	0.70	0.48	0.36	−0.14	−0.71	−0.55	−0.15	0.17	0.59
sub34	Val.	0.22	0.50	0.69	0.64	0.24	−0.07	−0.42	−0.46	−0.14
	Arou.	0.59	0.49	0.32	−0.22	−0.58	−0.44	−0.18	0.22	0.48
sub35	Val.	0.26	0.57	0.68	0.61	0.19	−0.18	−0.73	−0.47	−0.07
	Arou.	0.52	0.49	0.40	−0.20	−0.52	−0.48	−0.13	0.09	0.50
sub36	Val.	0.37	0.63	0.77	0.59	0.24	−0.15	−0.64	−0.60	−0.20
	Arou.	0.62	0.53	0.32	−0.27	−0.55	−0.40	−0.10	0.19	0.74
sub37	Val.	0.33	0.60	0.78	0.60	0.27	−0.07	−0.50	−0.54	−0.12
	Arou.	0.71	0.47	0.28	−0.23	−0.55	−0.45	−0.11	0.08	0.55
sub38	Val.	0.22	0.50	0.78	0.65	0.12	−0.11	−0.45	−0.37	−0.12
	Arou.	0.60	0.63	0.35	−0.19	−0.54	−0.38	−0.23	0.20	0.57
sub39	Val.	0.26	0.57	0.74	0.62	0.18	−0.10	−0.44	−0.47	−0.23
	Arou.	0.75	0.63	0.32	−0.32	−0.57	−0.53	−0.19	0.24	0.75
sub40	Val.	0.33	0.51	0.69	0.72	0.63	−0.29	−0.62	−0.50	−0.29
	Arou.	0.57	0.52	0.28	−0.24	−0.58	−0.46	−0.15	0.13	0.70
average	Val.	0.304	0.529	0.701	0.646	0.232	−0.122	−0.521	−0.470	−0.145
	Arou.	0.650	0.531	0.324	−0.210	−0.585	−0.476	−0.185	0.167	0.596
